# CINtec PLUS: A Novel Alternative Screening Method for Detecting High-Risk Cervical Lesions in Romania

**DOI:** 10.7759/cureus.69173

**Published:** 2024-09-11

**Authors:** Ovidiu A Camarasan, Andreea Camarasan, Mihaela M Muresan, Sorina Magheru, Andrei Pascalau, Andrea Pop-Crisan, Narcis Vilceanu, Ioana Vilceanu, Adrian Maghiar

**Affiliations:** 1 Department of Obstetrics and Gynecology, “Prof. Dr. Ioan Pușcaș” City Hospital of Șimleu Silvaniei, Oradea, ROU; 2 Department of Morphological Disciplines, Faculty of Medicine and Pharmacy, University of Oradea, Oradea, ROU; 3 Department of Anesthesiology, Faculty of Medicine and Pharmacy, University of Oradea, Oradea, ROU; 4 Department of Surgery Disciplines, Faculty of Medicine and Pharmacy, University of Oradea, Oradea, ROU

**Keywords:** asc-us, cintec plus, hpv, liquid based cytology, screening programme

## Abstract

Introduction

CINtec PLUS is a relatively recent method introduced in Romania for detecting cervical precancerous lesions. This technique utilizes simultaneous double immunostaining with p16 and Ki67 to identify potentially transformative human papillomaviruses (HPVs). CINtec PLUS has demonstrated superior sensitivity and specificity compared to conventional Papanicolaou (PAP) cytology and PAP liquid-based cytology, allowing for more accurate identification of HPV infections that may progress to malignancy among women with abnormal screening results. The objectives of this study are to evaluate CINtec PLUS test results in comparison with PAP cytology and HPV genotype detection among Romanian women and to assess its potential as a complementary screening method within existing national cervical cancer screening programs.

Materials and methods

Cases were collected between December 22, 2022, and June 15, 2024. All 96 women enrolled in the study, regardless of the presence of pathological cervical lesions, underwent the CINtec PLUS test. The samples were stained using the Roche CINtec PLUS kit. Statistical analyses were performed using IBM SPSS Statistics for Windows, Version 26.0 (Released 2019; IBM Corp., Armonk, NY, USA). To assess correlations and compare variables, we utilized crosstabulation tables, frequency tables, the chi-square test, and the Pearson correlation coefficient.

Results

The age range of participants in our study was from 19 to 64 years, with a mean age of 35.77 years and a standard deviation of 9.608. Among the women who underwent the CINtec PLUS test, over two-thirds were aged between 20 and 39 years. In 7.3% of cases with no history of HPV infection or cervical cytologic lesions, the CINtec PLUS test yielded a positive result. The study also revealed that CINtec PLUS showed a positive result in 23.3% of cases diagnosed with atypical squamous cells of undetermined significance on PAP cytology. Additionally, 36.5% of women who underwent CINtec PLUS testing as a screening method had a positive result, with more than one-fifth of these cases being positive.

Conclusions

The CINtec PLUS test is a valuable tool for identifying high-risk cervical lesions. Despite the limitations of our study, it provides a foundation for further research into the long-term benefits and cost-effectiveness of this test. Future studies could explore its potential for integration into national screening programs.

## Introduction

According to the WHO, cervical cancer affects over 60,000 women annually in Europe, with more than 30,000 deaths each year [1]. Despite being a preventable disease, cervical cancer still impacts approximately one in 111 women [1]. In Romania, cervical cancer ranks second in the European region, following Montenegro, with a mortality rate of 5-10 per 100,000 women [1-3].

Human papillomavirus (HPV) is the primary cause of nearly all cervical malignancies [1,4]. HPV is classified into high-risk (HR) and low-risk types based on its pathogenicity [5]. Persistent infection with HR HPVs can lead to cervical malignancies [6]. HPV types 16 and 18 account for almost 70% of cervical cancer cases [7,8]. Other oncogenic HR HPV types include 31, 33, 35, 39, 45, 51, 52, 56, 58, 68, 73, and 82 [7]. HPV16 is the most prevalent type globally, including in European countries and Romania [7]. Due to the high prevalence of HPV infection among Romanian women, screening programs have been developed and proven effective for diagnosing infections.

Premalignant cervical lesions associated with HPV are categorized as squamous intraepithelial lesions (SILs) or cervical intraepithelial neoplasia according to the latest WHO classification of female genital tumors [9]. The Bethesda system classifies cervical cell abnormalities into atypical squamous cells of undetermined significance (ASC-US), atypical squamous cells, cannot exclude high-grade SIL, low-grade SIL, high-grade SIL (H-SIL), and atypical glandular cells (AGCs) [10].

HPV infection can cause abnormalities in cell maturation and/or viral cytopathic effects that may lead to premalignant cervical lesions [9]. To diagnose these lesions, two primary methods are used: conventional Pap smears and liquid-based cytology (LBC) [11]. While both HPV detection and cervical cytology have significantly reduced morbidity and mortality worldwide, they each have limitations [12].

In Romania, both conventional Pap cytology and LBC are utilized. LBC offers advantages over conventional cytology by providing a thinner, monolayered smear. Samples are collected with a brush and placed into a liquid medium [13,14]. Recent data suggest that both methods have similar sensitivity for detecting premalignant lesions [14]. However, LBC slides are easier to analyze, and residual samples can be preserved for additional tests, such as CINtec PLUS and HPV detection [14].

CINtec PLUS, a relatively new method introduced in Romania, is used for detecting cervical precancerous lesions. It employs dual immunostaining with p16 and Ki67 to identify potentially transformative HPVs [12,15]. This test objectively identifies HPV infections that could progress to malignancy among women with abnormal screening results (Pap cytology and HPV detection) [15,16]. CINtec PLUS helps pathologists make informed recommendations and provides guidance for further evaluation, such as colposcopy [15].

Aim and scope

The primary aim of this study is to evaluate whether the CINtec PLUS test is superior to Pap cytology in detecting HR cervical lesions, particularly in patients diagnosed with ASC-US on Pap cytology. Additionally, the study explores the potential of CINtec PLUS as a future tool for cervical cancer screening management in Romania. It also investigates the correlation between HR HPV infections and the results of the CINtec PLUS test among women in Romania.

## Materials and methods

The prospective study was conducted at Resident Laboratory, a private facility located in western Romania. Resident Laboratory is the only private lab in this region offering the CINtec PLUS test and serves multiple counties, including Bihor, Arad, Timiș, and Sălaj. Private hospitals in the area have contracted Resident Laboratory for CINtec PLUS testing, whereas public hospitals in this region do not yet offer this test for managing precancerous cervical lesions.

Between December 22, 2022, and June 15, 2024, all women presenting for CINtec PLUS testing, regardless of their cytological status, were enrolled in the study. The Research Ethical Approval for the study was granted by the Ethics Committee of Resident Laboratory (approval number 2/25.07.2024), and written consent was obtained from all participants.

Inclusion criteria included all women who underwent CINtec PLUS testing during the specified time period, whether they had normal or abnormal cytology, including those who were tested as part of a screening program. Exclusion criteria were limited to unsatisfactory smears.

All women in the study underwent CINtec PLUS, a qualitative test that simultaneously detects p16INK4a and Ki67 proteins in cervical cytology samples. The procedure begins with clinicians, primarily gynecologists, collecting cervical samples using a thin brush. This brush is immediately placed into a liquid medium. The samples are then transported under proper conditions to the Resident Laboratory in Bihor County, Romania. If requested by the clinician, Pap LBC was also performed on the same sample.

At the laboratory, technicians process the samples, and experienced pathologists interpret the results. The samples, collected with a thin brush, were introduced into the Cyt-All ALPHAPATH solution according to the manufacturer’s instructions [17]. The Roche CINtec PLUS Cytology kit, a ready-to-use cocktail, includes monoclonal antibodies: p16 (E6H4™ mouse monoclonal) and Ki-67 (274-11 AC3 rabbit monoclonal). Quality control is performed using cervical carcinoma samples.

For a diagnosis of intraepithelial neoplasia, both biomarkers must be positive in the same cell. p16INK4a produces cytoplasmic and nuclear brown staining, while Ki67 stains the cell nucleus red. A positive CINtec PLUS result is indicated by the simultaneous double staining of a single cell. The samples were processed using the Ventana BenchMark GX system, following protocol instructions [18]. CINtec PLUS has recently been introduced in the United States as a new screening method for detecting HR cervical lesions [19].

All data, including both quantitative and categorical variables (nominal and ordinal), were entered into Microsoft Excel. Statistical analyses were conducted using IBM SPSS Statistics for Windows, Version 26.0 (Released 2019; IBM Corp., Armonk, NY, USA). For categorical variables, we employed crosstabulation tables, the chi-square test, Pearson correlation coefficient, and frequency tables. For quantitative variables, we calculated mean, median, and standard deviations. The positive and negative results of the CINtec PLUS test were compared with those from Pap cytology and HPV genotype detection. Statistical significance was determined using a p-value threshold of less than 0.05.

## Results

**Figure 1:**
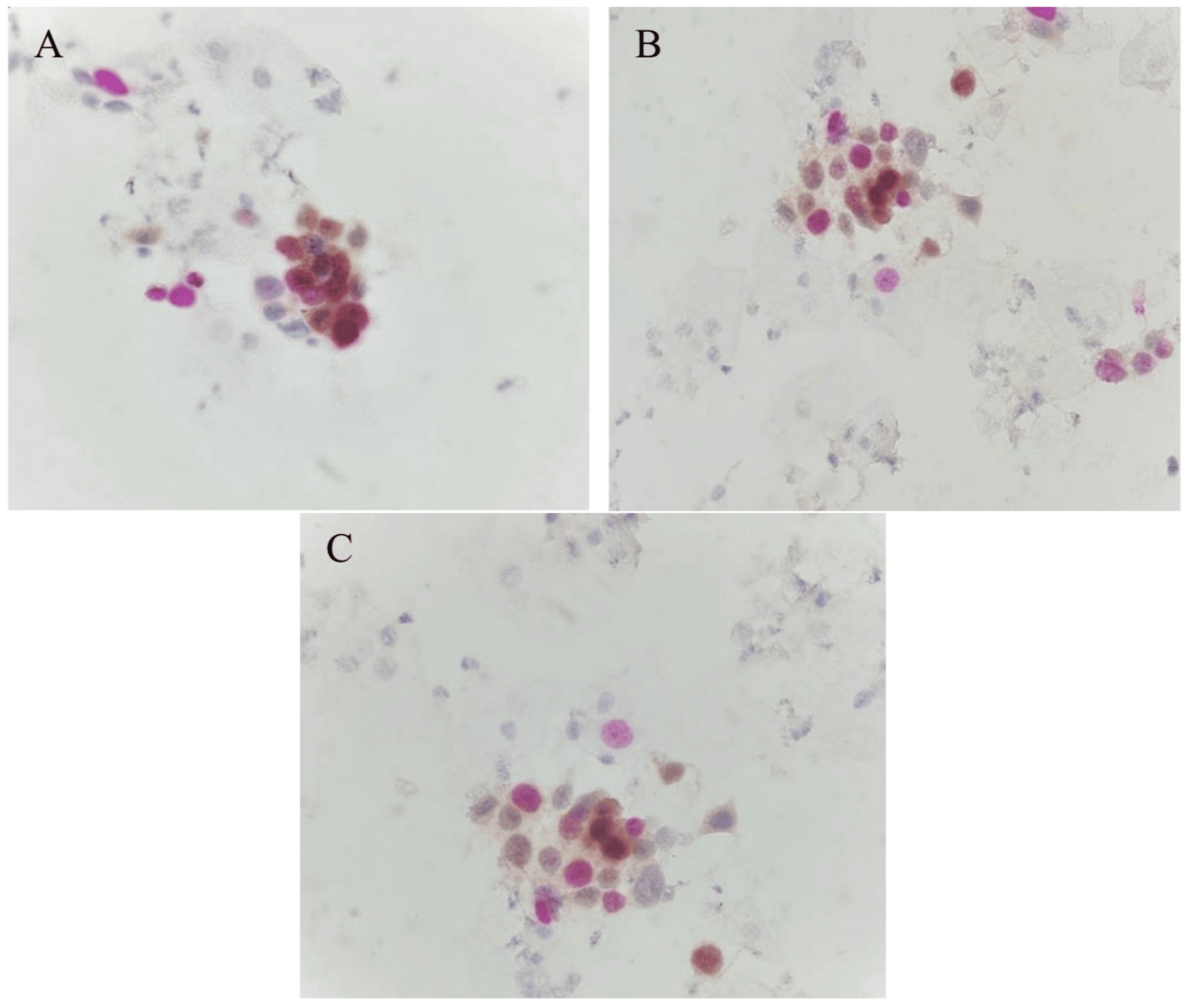
(A) OB40X, (B) OB20X, and (C) OB40X images show double immunostaining. p16 stains the cytoplasm brown, while Ki67 stains the nuclei red. The co-expression of both p16 and Ki67 in the same cell indicates cell cycle dysregulation, resulting in a positive interpretation of the CINtec PLUS test

For the CINtec PLUS test to be considered positive, indicating potential premalignant lesions, both immunomarkers must be positive in the same abnormal cell. In this test, p16 stains the cytoplasm and nucleus of the cervical cell brown, while Ki67, a nuclear marker, stains the abnormal nucleus red (Figure 1).

This study included 96 cases collected between December 22, 2022, and June 15, 2024. Participants ranged in age from 19 to 64 years, with a mean age of 35.77 years, a median age of 33 years, and a standard deviation of 9.608 years. The majority of participants were aged between 30 and 39 years (36.5%), followed by those aged 20 to 29 years (31.3%) (Table 1). Fewer participants were in the age groups under 20 years (1%) and over 60 years (1%). About one-third of the women underwent the CINtec PLUS test in conjunction with Pap LBC. The rest were referred for the CINtec PLUS test either due to a known cytological diagnosis or solely for screening purposes.

**Table 1:** Crosstabulation between age group and CINtec PLUS with or without PAP LBC PAP LBC, Papanicolaou liquid-based cytology

Age group			No PAP LBC with CINtec PLUS	PAP LBC and CINtec PLUS	Total
Age group	<20	Count	1	0	1
		% of total	1.00%	0.00%	1.00%
	20-29	Count	21	9	30
		% of total	21.90%	9.40%	31.30%
	30-39	Count	20	15	35
		% of total	20.80%	15.60%	36.50%
	40-49	Count	19	2	21
		% of total	19.80%	2.10%	21.90%
	50-60	Count	3	5	8
		% of total	3.10%	5.20%	8.30%
	>60	Count	0	1	1
		% of total	0.00%	1.00%	1.00%
Total		Count	64	32	96
		% of total	66.70%	33.30%	100.00%

There is a significant statistical association between the age groups and the cytological exams performed, as shown in Table 2 (chi-square test, p-value).

**Table 2:** Pearson chi-square correlation coefficient between age group and CINtec PLUS with or without PAP LBC ^a ^Five cells (41.7%) have an expected count of less than 5, with the minimum expected count being 0.33. PAP LBC, Papanicolaou liquid-based cytology

Test	Value	Df	Asymptotic significance (two sided)
Pearson chi-square	12.498^a^	5	0.029
Likelihood ratio	13.962	5	0.016
Linear-by-linear association	0.519	1	0.471
Number of valid cases	96	-	-

Although the finding is not statistically significant, it is noteworthy that out of the 21 cases with a positive CINtec PLUS result, 17 were in the age group of 20 to 39 years (Table 3).

**Table 3:** Crosstabulation between age group 20-39 years old and the result of CINtec PLUS

Result of the CINtec PLUS test (positive/negative)			Age group		Total
			20-39	Other age groups	
Result of CINtec PLUS	Negative	Count	48	27	75
		Expected count	50.8	24.2	75
		% within the age group	73.80%	87.10%	78.10%
	Positive	Count	17	4	21
		Expected count	14.2	6.8	21
		% within the age group	26.20%	12.90%	21.90%
Total		Count	65	31	96
		Expected count	65	31	96
		% within the age group	100.00%	100.00%	100.00%

Almost 25% (23 cases) of the cases were diagnosed with HR HPV infection, and the recommendation for the other patients with a positive CINtec PLUS result was to undergo HPV detection. Table 4 summarizes the frequencies for each HR HPV type and the crosstabulation for HR HPVs dependent on age group. The results showed that there is no statistical significance between age group and type of HPV (chi-square p-value 0.988). A total of 73 females underwent the CINtec PLUS exam without any known history of HPV infection.

**Table 4:** Crosstabulation between age groups and the type of HPV HR HPV, human papillomavirus; HR, high-risk

Type of HPV HR/no HPV known/unknown type of HPV HR		Age group						Total	Percentage
		<20	20-29	30-39	40-49	50-60	>60		
HPV HR	No HPV known	1	25	26	15	5	1	73	76.00%
	HPV18	0	0	2	1	1	0	4	4.20%
	HPV16	0	2	1	1	1	0	5	5.20%
	HPV31	0	0	1	0	0	0	1	1.00%
	HPV59	0	0	0	2	0	0	2	2.10%
	HPV82	0	1	0	0	0	0	1	1.00%
	HPV16 and HPV18	0	0	0	1	0	0	1	1.00%
	Unknown type of HPV HR	0	2	4	1	1	0	8	8.30%
	HPV56	0	0	1	0	0	0	1	1.00%
Total		1	30	35	21	8	1	96	100%

The most frequently met HR HPV type is HPV16, followed by HPV18. Table 5 shows the distribution of HR HPV types according to the result of the CINtec PLUS test. In 15 (15.6%) cases with no history of HPV infection, the CINtec PLUS result was positive. In the majority of cases where HPV16 and HPV18 were detected, the CINtec PLUS test result was negative (not statistically significant).

**Table 5:** Crosstabulation between the result of the CINtec PLUS test and the type of HPV HR HPV, human papillomavirus; HR, high-risk

Type of HPV HR/no HPV known/unknown type of HPV HR			Result of the CINtec PLUS test		Total
			Negative	Positive	
HPV HR	No HPV known	Count	58	15	73
		% of total	60.40%	15.60%	76.00%
	HR18	Count	3	1	4
		% of total	3.10%	1.00%	4.20%
	HR16	Count	4	1	5
		% of total	4.20%	1.00%	5.20%
	HR31	Count	0	1	1
		% of total	0.00%	1.00%	1.00%
	HR59	Count	2	0	2
		% of total	2.10%	0.00%	2.10%
	HR82	Count	1	0	1
		% of total	1.00%	0.00%	1.00%
	HR16&HR18	Count	1	0	1
		% of total	1.00%	0.00%	1.00%
	Unknown type of HPV HR	Count	6	2	8
		% of total	6.30%	2.10%	8.30%
	HR56	Count	0	1	1
		% of total	0.00%	1.00%	1.00%
Total		Count	75	21	96
		% of total	78.10%	21.90%	100.00%

More than two-thirds (75 cases) of all females had a negative CINtec PLUS result, with one-third of them not having PAP LBC associated with the CINtec PLUS method; 16 females without PAP LBC done at the same time as the p16/Ki67 double stain tested positive for CINtec PLUS, compared with just five females who tested positive for CINtec PLUS and had both tests performed at once (Table 6). However, the association of CINtec PLUS with PAP LBC is random, appeared by chance, and was statistically nonsignificant.

**Table 6:** Crosstabulation between the result of CINtec PLUS and the presence or absence of PAP LBC alongside CINtec PLUS PAP LBC, Papanicolaou liquid-based cytology

Result of the CINtec PLUS test (positive/negative)			Absence of PAP LBC	Presence of PAP LBC	Total
Result of the CINtec PLUS test	Negative	Count	48	27	75
		% of total	50.00%	28.10%	78.10%
	Positive	Count	16	5	21
		% of total	16.70%	5.20%	21.90%
Total		Count	64	32	96
		% of total	66.70%	33.30%	100.00%

We took into consideration the cytologic medical history results of each female. However, if the PAP LBC exam was performed alongside the CINtec PLUS exam, then the results of the last PAP LBC exam were considered prior to the cytologic medical history. The results show that ASC-US is the most frequently met cervical lesion diagnosed by PAP LBC or by conventional PAP smear. However, even if not statistically significant, our findings indicate that, although the majority of women were sent for CINtec PLUS examination due to ASC-US, only seven patients were diagnosed with a positive CINtec PLUS result, while the others had negative results. Out of the 6 cases sent to CINtec PLUS for H-SIL lesions, half were positive and the other half were negative (Table 7).

**Table 7:** Crosstabulation between PAP LBC and the result of CINtec PLUS AGC, atypical glandular cells; ASC-H, atypical squamous cells, cannot exclude high-grade squamous intraepithelial lesion; ASC-US, atypical cells of undetermined significance; H-SIL, high-grade squamous intraepithelial lesion; L-SIL, low-grade squamous intraepithelial lesion; Neg, negative; NILM, negative for intraepithelial lesion or malignancy; PAP LBC, Papanicolaou liquid-based cytology; Pos, positive

Lesions on cytology/no test known to be performed			Result of the CINtec PLUS test		Total
			Negative	Positive	
Lesions on cytology/no test known to be performed	Unknown/unperformed PAP LBC	Count	25	6	31
		% within CINtec Pos Neg	33.30%	28.60%	32.30%
	NILM	Count	11	0	11
		% within CINtec Pos Neg	14.70%	0.00%	11.50%
	ASC-US	Count	23	7	30
		% within CINtec Pos Neg	30.70%	33.30%	31.30%
	ASC-H	Count	5	3	8
		% within CINtec Pos Neg	6.70%	14.30%	8.30%
	L-SIL	Count	5	1	6
		% within CINtec Pos Neg	6.70%	4.80%	6.30%
	H-SIL	Count	3	3	6
		% within CINtec Pos Neg	4.00%	14.30%	6.30%
	AGC	Count	3	1	4
		% within CINtec Pos Neg	4.00%	4.80%	4.20%
Total		Count	75	21	96
		% within CINtec PLUS	100.00%	100.00%	100.00%

A positive result of the CINtec PLUS test in women with ASC-US lesions diagnosed by PAP cytology was observed in just 23.3%, statistically nonsignificant. This result means that most of the patients diagnosed with ASC-US on conventional PAP smear or on PAP LBC are most likely to have a negative result on the CINtec PLUS exam; therefore, ASC-US is a cytological diagnostic that in most cases does not lead to precancerous cervical lesions (Table 8).

**Table 8:** Crosstabulation between the presence/absence of ASC-US on cytology and the result of CINtec PLUS ASC-US, atypical squamous cells of undetermined significance

Result of the CINtec PLUS test (positive/negative)			ASC-US		Total
			Yes	No	
Result of the CINtec PLUS test	Positive	Count	7	14	21
		% within ASC-US	23.30%	21.20%	21.90%
	Negative	Count	23	52	75
		% within ASC-US	76.70%	78.80%	78.10%
Total		Count	30	66	96
		% within ASC-US	100.00%	100.00%	100.00%

Thirty-five (36.5%) out of 96 females applied for the CINtec PLUS test as a screening method, and more than one-fifth were positive for the p16/Ki67 double immunostaining (Table 9). Although the positive result of the CINtec PLUS test is not high (22.9%, statistically nonsignificant), our study indicates that more than one-fifth of all females who underwent the CINtec PLUS test for screening purposes are potentially at risk for HR cervical lesions.

**Table 9:** Crosstabulation between the result of CINtec PLUS and the presence/absence of screening

Result of the CINtec PLUS test (positive/negative)			Screening		Total
			Yes	No	
Result of the CINtec PLUS test	Positive	Count	8	13	21
		% within yes or no screening	22.90%	21.30%	21.90%
	Negative	Count	27	48	75
		% within yes or no screening	77.10%	78.70%	78.10%
Total		Count	35	61	96
		% within yes or no screening	100.00%	100.00%	100.00%

## Discussion

One of the most important screening methods for cervical lesions in Romania remains the detection of HR HPV, as it is the primary cause of cervical precancerous lesions that could transform into cervical cancer. This method has significantly reduced mortality and morbidity among women in Romania in recent years. However, although HPV infection resolves on its own in the majority of cases, there are instances where it becomes persistent despite medical treatment, potentially causing precancerous cervical lesions [20]. A study conducted by Simion et al. in Romania highlighted the importance of HPV screening programs among women and the need for their continuity, as the prevalence of HPV remains high in this country [21]. Clinicians need to find out not only if a woman has been infected with HPV but also if HPV has produced cervical lesions. To meet this need, another screening program, the PAP test, was developed. In the west of Romania, a conventional PAP smear program is running for women aged between 24 and 29 years old; for women aged between 30 and 64 years old, HPV detection was the first step of the screening program, in case a positive result of PAP LBC was performed. Regarding accessibility to cervical screening programs, studies show that in Romania, discrepancies still exist between females from rural areas and those from urban areas, and the mortality rate due to cervical cancer is 24% higher in women from rural regions compared to those from urban regions [21]. Although the national screening programs are accessed by female populations, unfortunately, in some cases, the current existing screening methods can misdiagnose or even overdiagnose precancerous lesions. In these situations, an alternative with higher specificity compared with HPV detection in terms of HR cervical lesions available in Romania is CINtec PLUS.

Young reproductive females are the most predisposed group to infection of HPV, as HPV is a sexually transmitted disease [22,23]. In Romania, according to our study, the median age for women who underwent CINtec PLUS examination is 33, which is similar to the most recent data from a study in the United States that demonstrated that the median age in the female population was 33.5 [24]. According to scientific data, HPV16 is the most frequently met HR type among women from Romania, aged between 31 and 40 years old [23,25]. These findings are also supported by our results, which show that the most frequently met HR HPVs are HPV16, followed by HPV18. Another HR HPV type listed in our study is HPV56, with a higher prevalence compared with HPV31, HPV56, and HPV82. The prevalence of HPV16, HPV31, HPV51, HPV18, HPV52, and HPV58, in descending order, is reported as the most frequently encountered HR HPV types in the central region of Romania, specifically in Brașov County, according to a study conducted by Moga et al. [26]. Despite the diversity of HR HPV types being found among women in Romania, HPV16 remains in the top position of the ranking.

It is known that a positive CINtec PLUS result is associated with H-SIL cervical lesions or with invasive carcinoma [27]. If Ki67, a proliferation marker, and p16, considered a tumor suppressor protein, are simultaneously double-stained in the same cells, then the cervical cells have undergone HPV-associated transformations [27]. Because CINtec PLUS was performed in both situations: as a triage method and as a result of abnormal cytological results, the test showed positive results in one-third of all our cases. A study conducted by Sharma et al. showed a positive CINtec PLUS test in more than two-thirds of the studied group [12]. However, in that study, only females diagnosed with squamous epithelial cell abnormalities were included, compared to our study, which included also patients without any history of abnormal cervical lesions [12]. The positive result of the CINtec PLUS test was observed in 22.8% of all females who underwent this test as a screening method. This percentage is small, but in clinical practice, if 22 out of 100 women have a positive result on the CINtec PLUS test, it means that further examinations need to be done because each of these women is more likely to have HR cervical intraepithelial lesions.

In those cases in which ASC-US was the reason for sending the patient for a CINtec PLUS test, our study demonstrated that usually this condition does not lead to precancerous cervical lesions; more than 75% of the cases with ASC-US on PAP LBC had a negative result on the CINtec PLUS test. A retrospective study conducted in Belgium by Cras et al. supports our findings, with the CINtec PLUS test showing negative results in 83% of the cases [27]. Taking these results into consideration, we can acknowledge that ASC-US is usually a benign condition, but sometimes the CINtec PLUS test result can be positive, indicating the need for correct management and treatment for those patients.

Our study revealed that from the total of the H-SIL lesions diagnosed by PAP cytology, only half of them had a positive result on the CINtec PLUS test. The result is low compared with a study conducted by Cras et al. in Belgium but is expected, considering that not all women with H-SIL lesions underwent CINtec PLUS examination alongside PAP LBC [27]. For half of them, women who were known to have H-SIL lesions in their medical history, the CINtec PLUS exam was performed as a method of preventive care to determine if the lesion still exists.

Thus, early diagnosis is of great importance in cancerous lesions. In the case of precancerous lesions, in addition to clinical follow-up, increasing the amount of antioxidants can be taken into account [28].

The limitations of the study are primarily represented by the lack of histological cervical results (results of the biopsies). Out of all the females enrolled in this study, only three biopsy results were available for the women who had positive results for the CINtec Plus test and were also diagnosed with H-SIL on PAP LBC. According to the American Society for Colposcopy and Cervical Pathology (ASCCP) guidelines, women diagnosed with ASC-US or NILM on PAP LBC, without any other cytologic history and with a current positive or negative HPV result, require a five-year follow-up, three-year follow-up, or a one-year follow-up in certain cases [29]. In women without a prior cytologic history but with a current HR HPV infection and a current ASC-US result on cytology, colposcopy is the recommended management. Considering the results of our study, in which we evaluated the CINtec Plus test results, recommendations regarding colposcopy/treatment were made when H-SIL was diagnosed on current PAP LBC. Other limitations of this study are represented by the small number of cases, despite the large time interval in which they were gathered. A possible reason for the small sample size is the fact that this method is more expensive compared with conventional PAP, and the financial cost is not supported by the Ministry of Health in Romania. Not all patients underwent PAP LBC alongside CINtec PLUS. Some patients had a medical cervical history, while others came without any medical history; they came exclusively for screening purposes.

When applied correctly, the CINtec PLUS test can reduce the repeated number of colposcopies and biopsies, reducing the costs and increasing the benefits for the patients. According to a recent study performed in the United States, the CINtec PLUS test, which was recently accepted as a new screening method for HR cervical lesions, along with HPV genotype detection and cervical cytology, is expected to be cost-effective [30]. At the moment, in a middle-income country like Romania, where public hospitals cover the cost of minimally invasive procedures, women are more likely to opt for financially covered procedures rather than a payable method, such as CINtec PLUS, at a private hospital. The solution to this problem is to introduce the CINtec PLUS test, a method of screening among women in Romania for more accurate and individualized patient management. For supplementary data and more conclusive results regarding CINtec PLUS as a method of screening, a national study needs to be performed, and the study above can be considered a trigger for further research.

## Conclusions

Romania continues to have one of the highest rates of cervical cancer in Europe, highlighting the need for improved national screening programs to detect precancerous cervical lesions. The CINtec PLUS test offers a promising alternative for identifying HR cervical lesions, demonstrating higher specificity than HPV detection and greater sensitivity than cytology. Despite its limitations, our study serves as an initial step toward larger, future research. Further evaluation of CINtec PLUS is needed to assess its potential as a national screening tool for HR cervical lesions, focusing on cost-effectiveness and long-term benefits for both patients and clinicians.
